# Proximity-dependent protein (de)stabilization: screening the human ORFeome for protein degraders and stabilizers

**DOI:** 10.1038/s41392-024-01884-3

**Published:** 2024-07-26

**Authors:** Thomas Hermanns, Kay Hofmann

**Affiliations:** Institute for Genetics, University of Cologne, Cologne, Germany

**Keywords:** Target identification, Target identification

In a recent study published in *Nature*, Poirson et al.^1^ report an unbiased screen for proteins that can either bring about—or prevent—the destruction of proteinaceous model substrates in a proximity-dependent manner. Knowledge of such protein degraders and stabilizers will be instrumental in the development of new drug classes that specifically regulate the abundance of their target proteins.

The most widely used application of targeted protein degradation (TPD) uses so-called PROTACs (proteolysis-targeting chimeras), bifunctional small molecules that can simultaneously bind to a ubiquitin ligase (E3) and a substrate protein.^2^ In response to induced proximity, a degradative ubiquitin chain is installed on the substrate, leading to its degradation by the proteasome. To work in a PROTAC setting, a ubiquitin ligase must be sufficiently abundant in the target cells, generate the right kind of ubiquitin chains (usually K48-linked) on proximal substrates without much intrinsic specificity, and possess a suitable small-molecule binding pocket. This last condition is a major bottleneck restricting the number of actively used ligases to a handful, of which CRBN (Cereblon) and VHL (von Hippel–Lindau disease protein) are by far the most prominent ones.^3^ In their unbiased primary screen, the authors used an eGFP-ABI1 fusion protein as a model substrate, which was expressed together with a non-targeted fluorescent protein (BFP) from a bicistronic vector. Effectors were targeted towards eGFP-ABI1 either by fusing them to a GFP-directed nanobody or by fusing them to PYL1, a plant protein that can be chemically induced to bind to the ABI1 moiety of the substrate. The eGFP-ABI1 (de)stabilization was measured using the eGFP/BFP ratio. Most identified effectors with strong destabilizing effects were indeed ubiquitin ligases or components of ligase complexes. Notably, VHL was not found in the top group, while CRBN was not covered by the screening library. The aptitude of ubiquitin ligases for TPD was further underscored by a second screen, in which 300 human ligases were targeted to eGFP-ABI1 by fusing them to an anti-GFP nanobody. Approximately half of the tested ligases significantly decreased the eGFP/BFP ratio relative to that of the negative control. Overall, there was good concordance with the primary screen. Both VHL and CRBN showed strong destabilizing effects, but many other RING-E3s and cullin adaptors showed similar or even stronger effects. The tested U-box ligases were surprisingly ineffective, whereas HECT and RBR-ligases were not included in the screen, probably owing to their unwieldy size. An ideal TPD ligase should be able to destabilize a wide range of substrates and show little dependence on linker length or position. Several of the top candidates identified in the screens fulfilled these criteria. When tested against ten model substrates with different subcellular localizations, FBXL12, FBXL14, FBXL15, KBTBD7, and PRAME destabilized most of them, whereas CRBN and particularly VHL were more restrictive.

Interestingly, the unbiased screen for destabilizing factors did not only yield ubiquitin ligases. A strong and consistent effect was observed for the ubiquitin-conjugating (E2) enzyme UBE2B, which was not dependent on its E3-binding interface. In a targeted screen, this destabilizing activity was shared by 13 of the 30 tested human E2 enzymes, suggesting that E2s are a largely untapped resource for future TPD efforts.^4^ Some E2s did not destabilize the model substrate or even increased its stability, possibly because of their different linkage propensities. Other proteins with less obvious links to ubiquitination were also found among the destabilizing effectors. Some of these, including several LC3-like proteins, are involved in autophagy and accordingly lost their destabilization effect upon inhibition of the autophagy kinases ULK1/2. Others, including PRR20A and EID1, are known or assumed to contain strong degrons and probably mediate TPD in a more indirect manner.

**Fig. 1:**
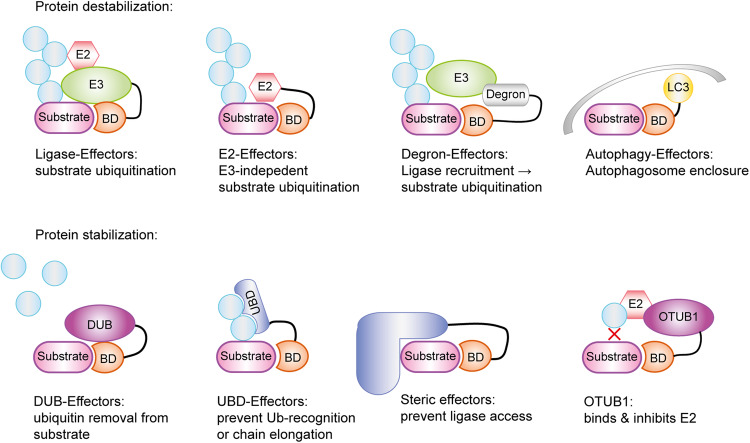
Schematic overview of protein (de)-stabilizing mechanisms. Poirson et al.^1^ identified several effectors in their unbiased screen that either degrade or stabilize a model substrate. These effectors are fused to a binding domain (BD) directed against a model substrate and can be grouped based on their mechanism. Ubiquitin ligases (E3) and conjugating enzymes (E2) directly ubiquitinate (light blue circles) the substrate protein, leading to its proteasomal degradation. In at least one example, the E2 enzyme was shown to function without E3. Degrons fused to the BD recruit E3 ligases into close proximity to the substrate, thereby indirectly causing its ubiquitination. Alternatively, LC3-like destabilizers can recruit phagophore membranes (gray structure) and target the substrate for autophagy. Conversely, deubiquitinases (DUBs) remove ubiquitin chains from the substrate, leading to its stabilization. Steric and UBD effectors function by preventing further ubiquitination or ubiquitin recognition by limiting access to the required factors. A special case identified by Poirson et al. is the DUB OTUB1, whose stabilizing effect was shown to be dependent on its ability to bind and inhibit E2 enzymes

The unbiased screen performed by Poirson et al. also identified stabilizing effectors, which might be useful for future therapeutic interventions aimed at preventing the degradation of tumor suppressors or proteins rendered unstable by pathogenic mutations. A prominent example of targeted protein stabilization (TPS) is the recently published application of deubiquitinase-targeting chimeras (DUBTACs), which target the K48-specific deubiquitinase OTUB1 to the unstable ΔF508 mutant of the cystic fibrosis transmembrane conductance regulator CFTR.^5^ It is assumed—while not rigorously proven—that proximity to an active deubiquitinase causes the removal of degradative ubiquitin chains, thus sparing the target from proteasomal degradation. However, the generality of this assumption is not supported by the data from Poirson et al. First, the unbiased screen for highly stabilizing effectors yielded few deubiquitinases, with OTUB1, UCHL1, and OTUD6 as the only notable examples. Moreover, a second screen testing 47 selected deubiquitinases, targeted via a nanobody to an eGFP-coupled unstable reporter protein, yielded only nine significantly stabilizing DUB effectors, including OTUB1, USP39, and USP13. Surprisingly, the stabilizing function of OTUB1 did not depend on its active site, while USP39 is an inactive pseudoenzyme, and active site mutants of USP13 still conferred partial stabilization. Further validation by the authors revealed that the ubiquitin-binding domains (UBDs) of these enzymes contribute most to the stabilization effect, while the known E2-inhibiting function of OTUB1 is also important. The relatively short linkers used in the screen may have prevented deubiquitinase effectors from reaching all ubiquitinated residues of the substrate, thus impeding canonical DUBTAC activity. Nevertheless, the strong stabilizing effects seen upon proximity with ubiquitin-binding domains hold promise for the future development of “UBDTACs” as an alternative TPS modality. Beyond DUBs and UBDs, the KLH40 protein showed particularly strong and consistent stabilization. As a BTB-domain protein, KLHL40 would be expected to destabilize proximal proteins by interacting with the CUL3 ligase complex. However, KLHL40 lacks a residue important for cullin interaction and was found to stabilize a wide range of model substrates with little dependence on linker length or position. Its mode of action is currently unclear and may involve steric shielding of its proximity partner. Like any good study, the work by Poirson et al. not only answers important questions but also raises new ones. Doubtlessly, these new insights will impact the future of targeted protein (de)stabilization research (Fig. 1).
